# Supplementary material for: Transcriptome profiles of Trypanosoma brucei rhodesiense in Malawi reveal focus specific gene expression profiles associated with pathology
Source: PLoS Negl Trop Dis. 2024 May 3;18(5):e0011516. doi: 10.1371/journal.pntd.0011516 (PMC11095692; doi:10.1371/journal.pntd.0011516)
Supplement: S1 Table — brucei rhodesiense isolates from Nkhotakota focus versus Rumphi focus. (DOCX) [file pntd.0011516.s001.docx]

**Table S1**: List of Genes that were significant (padj<0.05) differentially expressed in *T. brucei rhodesiense* isolates from Nkhotakota focus versus Rumphi focus.

| **Gene_ID** | **Gene Name** | **padj** | **NKHOTAKOTA_log2FC** | **RUMPHI_log2FC** |
| --- | --- | --- | --- | --- |
| Tb927.1.5220:mRNA | expression site-associated gene 9 (ESAG9) protein | 8.57E-14 | 7.0 | -7.0 |
| Tb09.v4.0065:mRNA | expression site-associated gene 1 (ESAG 1) | 5.39E-17 | 5.1 | -5.1 |
| Tb927.11.17890:mRNA | expression site-associated gene 1 (ESAG1) protein | 3.93E-10 | 4.5 | -4.5 |
| Tb927.9.680:pseudogenic_transcript | expression site-associated gene 9 (ESAG9) | 2.43E-09 | 4.1 | -4.1 |
| Tb927.11.17870:mRNA | variant surface glycoprotein (VSG) | 1.62E-12 | 4.0 | -4.0 |
| Tb927.9.700:mRNA | hypothetical protein | 0.03518301 | 3.3 | -3.3 |
| Tb927.11.18670.1 | expression site-associated gene 9 (ESAG9) | 0.00354186 | 3.2 | -3.2 |
| Tb927.9.690:pseudogenic_transcript | variant surface glycoprotein (VSG | 3.03E-05 | 3.2 | -3.2 |
| Tb11.1000:mRNA | expression site-associated gene 9 (ESAG9) protein | 0.02298155 | 3.2 | -3.2 |
| Tb927.9.660:mRNA | expression site-associated gene 1 (ESAG1) protein | 0.00033973 | 3.0 | -3.0 |
| Tb11.v5.0984.1 | hypothetical protein | 0.00753467 | 3.0 | -3.0 |
| Tb9.NT.6:ncRNA | Noncoding RNA | 0.00139569 | 2.6 | -2.6 |
| Tb927.5.150:mRNA | hypothetical protein | 0.02298155 | 2.4 | -2.4 |
| Tb927.1.4900:mRNA | expression site-associated gene 11 (ESAG11) protein | 0.01320193 | 1.9 | -1.9 |
| Tb927.2.510:mRNA | retrotransposon hot spot protein 4 (RHS4) | 1.10E-06 | 1.9 | -1.9 |
| Tb927.7.370:mRNA | hypothetical protein | 0.00945914 | 1.5 | -1.5 |
| Tb927.1.4910:mRNA | expression site-associated gene 1 (ESAG1) protein | 0.0001782 | 1.5 | -1.5 |
| Tb11.0290:mRNA | chrXI additional | 0.02315865 | 1.3 | -1.3 |
| Tb927.7.410:mRNA | hypothetical protein | 0.02655349 | 1.2 | -1.2 |
| Tb927.2.3310:mRNA | 65 kDa invariant surface glycoprotein | 0.03022022 | 1.1 | -1.1 |
| Tb927.4.3550:mRNA | 60S ribosomal protein L13a | 0.04148499 | 1.0 | -1.0 |
| Tb927.11.10740:mRNA | vacuolar sorting-associated protein-like | 0.04606608 | 0.9 | -0.9 |
| Tb927.10.560:mRNA | 40S ribosomal proteins S11 | 0.04148499 | 0.9 | -0.9 |
| Tb927.5.620:mRNA | invariant surface glycoprotein | 0.0289159 | 0.9 | -0.9 |
| Tb11.v5.0871.1 | hypothetical protein | 0.02068972 | 0.9 | -0.9 |
| Tb927.9.14000:mRNA | 60S ribosomal protein L12 | 0.02343717 | 0.8 | -0.8 |
| Tb11.v5.0326.1 | retrotransposon hot spot (RHS) protein | 0.04148499 | 0.7 | -0.7 |
| Tb927.3.1290:mRNA | cullin 4B | 0.04148499 | -0.7 | 0.7 |
| Tb927.1.180:mRNA | retrotransposon hot spot protein 1 (RHS1) | 0.04325465 | -0.7 | 0.7 |
| Tb927.11.16010:mRNA | hypothetical protein | 0.04325465 | -0.7 | 0.7 |
| Tb927.11.4280:mRNA | hypothetical protein | 0.04264455 | -0.8 | 0.8 |
| Tb927.9.12160:mRNA | hypothetical protein | 0.02286025 | -0.8 | 0.8 |
| Tb927.11.890:mRNA | hypothetical protein | 0.01394418 | -0.8 | 0.8 |
| Tb927.8.6790:mRNA | hypothetical protein | 0.01149439 | -0.8 | 0.8 |
| Tb927.1.220:mRNA | retrotransposon hot spot protein 1 (RHS1) | 0.01394418 | -0.8 | 0.8 |
| Tb927.1.120:mRNA | retrotransposon hot spot protein 4 (RHS4) | 0.03324446 | -0.8 | 0.8 |
| Tb927.10.14510:mRNA | root hair defective 3 GTP-binding protein (RHD3) | 0.00446131 | -0.8 | 0.8 |
| Tb927.10.14900:mRNA | hypothetical protein | 0.04738524 | -0.9 | 0.9 |
| Tb927.9.5520:mRNA | ubiquitin carboxyl-terminal hydrolase | 0.00615567 | -0.9 | 0.9 |
| Tb927.7.6650:mRNA | Colon cancer-associated protein Mic1-like | 0.0328617 | -0.9 | 0.9 |
| Tb927.11.6120:mRNA | ABC transporter | 0.02640014 | -0.9 | 0.9 |
| Tb927.7.6760.1:mRNA | hypothetical protein | 0.00945914 | -0.9 | 0.9 |
| Tb927.2.5480:mRNA | hypothetical protein | 0.04577389 | -0.9 | 0.9 |
| Tb927.10.2570:mRNA | lysosomal alpha-mannosidase precursor | 0.00229187 | -0.9 | 0.9 |
| Tb11.02.5130b.1 | neurobeachin/beige protein | 0.04148499 | -0.9 | 0.9 |
| Tb927.4.4510:mRNA | protein phosphatase 2C | 0.04148499 | -1.0 | 1.0 |
| Tb927.1.5150:mRNA | hypothetical protein | 0.00250144 | -1.0 | 1.0 |
| Tb927.10.11470:mRNA | hypothetical protein | 0.03264334 | -1.0 | 1.0 |
| Tb927.5.2360:mRNA | hypothetical protein | 0.04444358 | -1.1 | 1.1 |
| Tb927.11.810:mRNA | hypothetical protein | 0.02286025 | -1.1 | 1.1 |
| Tb927.4.5350:pseudogenic_transcript | 3-methylcrotonyl-CoA carboxylase | 0.02286025 | -1.1 | 1.1 |
| Tb11.v5.1039.1 | nuclear cap binding complex subunit CBP110 | 0.00803627 | -1.1 | 1.1 |
| Tb11.1200:mRNA | hypothetical protein | 0.04606608 | -1.1 | 1.1 |
| Tb927.7.6780:mRNA | hypothetical protein | 0.04947362 | -1.1 | 1.1 |
| Tb927.10.2990:mRNA | nuclear cap binding complex subunit CBP110 | 0.00160073 | -1.1 | 1.1 |
| Tb927.1.4870:mRNA | expression site-associated gene 1 (ESAG1) protein | 0.04148499 | -1.2 | 1.2 |
| Tb927.8.7830:mRNA | hypothetical protein | 0.00097027 | -1.3 | 1.3 |
| Tb927.1.2880:mRNA | pteridine transporter | 0.0328617 | -1.3 | 1.3 |
| Tb927.2.240:mRNA | retrotransposon hot spot protein 5 (RHS5) | 0.02612907 | -1.3 | 1.3 |
| Tb927.7.7540:pseudogenic_transcript | leucine-rich repeat protein 1 (LRRP1) | 0.00076855 | -1.4 | 1.4 |
| Tb927.7.3830:mRNA | kinesin K39 | 0.01728125 | -1.4 | 1.4 |
| Tb11.v5.0301.1 | glycerol uptake protein | 7.46E-05 | -1.5 | 1.5 |
| Tb927.6.550:mRNA | hypothetical protein | 0.0027805 | -1.6 | 1.6 |
| Tb927.10.12750:mRNA | hypothetical protein | 0.01269153 | -1.6 | 1.6 |
| Tb09.v4.0109:mRNA | hypothetical protein | 0.0327634 | -1.7 | 1.7 |
| Tb11.v5.0381.1 | hypothetical protein | 0.00346162 | -1.7 | 1.7 |
| Tb11.v5.0416.1 | Variant Surface Glycoprotein | 0.04947362 | -1.8 | 1.8 |
| Tb11.1470:pseudogenic_transcript | variant surface glycoprotein (VSG | 0.04161627 | -1.8 | 1.8 |
| Tb09.v4.0143:pseudogenic_transcript | variant surface glycoprotein (VSG | 0.0327634 | -1.9 | 1.9 |
| Tb927.4.240:pseudogenic_transcript | retrotransposon hot spot protein 4 (RHS4) | 0.01563118 | -1.9 | 1.9 |
| Tb927.1.5170:mRNA | variant surface glycoprotein (VSG)-related | 8.91E-06 | -2.0 | 2.0 |
| Tb927.1.5110:mRNA | expression site-associated gene 11 (ESAG11) protein | 0.00250144 | -2.0 | 2.0 |
| Tb927.7.6540:mRNA | variant surface glycoprotein (VSG | 0.04161627 | -2.3 | 2.3 |
| Tb10.v4.0096:mRNA | variant surface glycoprotein (VSG) | 0.03139194 | -2.4 | 2.4 |
| Tb927.11.17460:mRNA | variant surface glycoprotein (VSG) | 0.04090295 | -2.5 | 2.5 |
| Tb927.9.15560:mRNA | BARP protein | 0.0079104 | -2.7 | 2.7 |
| Tb927.11.17540:pseudogenic_transcript | variant surface glycoprotein (VSG | 0.00346162 | -2.7 | 2.7 |
| Tb11.v5.0376.1 | Trypanosomal VSG domain containing protein | 0.00346162 | -2.7 | 2.7 |
| Tb927.4.200:mRNA | retrotransposon hot spot protein 1 (RHS1) | 4.49E-08 | -3.0 | 3.0 |
| Tb927.10.10220:mRNA | NA | 0.00630177 | -3.2 | 3.2 |
| Tb927.3.5820:pseudogenic_transcript | expression site-associated gene 11 (ESAG11) | 1.19E-05 | -3.5 | 3.5 |
| Tb08.27P2.80:mRNA | hypothetical protein | 0.00179787 | -4.2 | 4.2 |
| Tb927.6.5320:pseudogenic_transcript | NA | 0.04264455 | -4.3 | 4.3 |
| Tb11.1060:pseudogenic_transcript | NA | 0.04161627 | -4.5 | 4.5 |
| Tb927.11.17550:mRNA | NA | 0.00160073 | -4.5 | 4.5 |
| Tb10.v4.0070:mRNA | NA | 0.02405468 | -4.7 | 4.7 |
| Tb927.1.5240:mRNA | NA | 0.02308413 | -4.8 | 4.8 |
| Tb08.27P2.70:mRNA | NA | 0.00179787 | -5.3 | 5.3 |
| Tb927.6.182:rRNA | M6 ribosomal RNA | 3.37E-21 | -5.4 | 5.4 |
| Tb10.v4.0061:pseudogenic_transcript | NA | 0.00615567 | -5.8 | 5.8 |
