# Supplementary material for: Transcriptome profiles of Trypanosoma brucei rhodesiense in Malawi reveal focus specific gene expression profiles associated with pathology
Source: PLoS Negl Trop Dis. 2024 May 3;18(5):e0011516. doi: 10.1371/journal.pntd.0011516 (PMC11095692; doi:10.1371/journal.pntd.0011516)
Supplement: S2 Table — rhodesiense biological processes of differentially enriched genes (DEGs) that were upregulated (log2FC > 1) in Nkhotakota focus and loaded in TritrypDB. The fold enrichment is the percentage of genes loaded divide by the percentage of genes with this term in the background. The p-value measured the Fishers exact test. (DOCX) [file pntd.0011516.s002.docx]

**Table S2:** Significant (p<0.05) gene ontology enrichment of *T.b. rhodesiense* biological processes of differentially enriched genes (DEGs) that were upregulated (log2FC > 1) in Nkhotakota focus and loaded in TritrypDB. The fold enrichment is the percentage of genes loaded divide by the percentage of genes with this term in the background. The p-value measured the Fishers exact test.

| Gene Ontology | Biological Process | Fold Enrichment (FE) | P-value of FE |
| --- | --- | --- | --- |
| GO:0010256 | Endomembrane system organization | 52.9 | 2.09E-05 |
| GO:0006890 | Retrograde vesicle-mediated transport, Golgi to endoplasmic reticulum | 80.32 | 0.000265 |
| GO:0007030 | Golgi organization | 49.86 | 0.000698 |
| GO:0048193 | Golgi vesicle transport | 16.06 | 0.006566 |
| GO:0007009 | Plasma membrane organization | 103.27 | 0.009646 |
| GO:0046907 | Intracellular transport | 5.71 | 0.013654 |
| GO:0033036 | macromolecule localization | 5.63 | 0.014146 |
| GO:0051649 | establishment of localization in cell | 5.6 | 0.014345 |
| GO:0071850 | mitotic cell cycle arrest | 60.24 | 0.016484 |
| GO:0071705 | nitrogen compound transport | 5.21 | 0.017424 |
| GO:0009299 | mRNA transcription | 51.64 | 0.019207 |
| GO:0042789 | mRNA transcription by RNA polymerase II | 51.64 | 0.019207 |
| GO:0051641 | cellular localization | 4.99 | 0.019631 |
| GO:0016043 | cellular component organization | 3.52 | 0.021239 |
| GO:0071702 | organic substance transport | 4.82 | 0.021481 |
| GO:0000028 | ribosomal small subunit assembly | 40.16 | 0.024632 |
| GO:0034249 | negative regulation of cellular amide metabolic process | 34.42 | 0.028683 |
| GO:0017148 | negative regulation of translation | 34.42 | 0.028683 |
| GO:0006406 | mRNA export from nucleus | 26.77 | 0.036739 |
| GO:0051248 | negative regulation of protein metabolic process | 25.82 | 0.038075 |
| GO:0032269 | negative regulation of cellular protein metabolic process | 25.82 | 0.038075 |
| GO:0016192 | vesicle-mediated transport | 6.29 | 0.038778 |
| GO:0071840 | cellular component organization or biogenesis | 2.92 | 0.039403 |
| GO:0006886 | intracellular protein transport | 6.15 | 0.040331 |
| GO:0000462 | maturation of SSU-rRNA from tricistronic rRNA transcript (SSU-rRNA, 5.8S rRNA, LSU-rRNA) | 24.1 | 0.040744 |
| GO:0051028 | mRNA transport | 23.32 | 0.042075 |
| GO:0034645 | cellular macromolecule biosynthetic process | 3.68 | 0.04321 |
| GO:0009059 | macromolecule biosynthetic process | 3.66 | 0.04377 |
| GO:0006405 | RNA export from nucleus | 21.26 | 0.04606 |
