# Supplementary material for: Transcriptome profiles of Trypanosoma brucei rhodesiense in Malawi reveal focus specific gene expression profiles associated with pathology
Source: PLoS Negl Trop Dis. 2024 May 3;18(5):e0011516. doi: 10.1371/journal.pntd.0011516 (PMC11095692; doi:10.1371/journal.pntd.0011516)
Supplement: S3 Table — rhodesiense biological processes of differentially enriched genes (DEGs) that were upregulated (log2FC > 1) in Rumphi focus and loaded in TritrypDB. The fold enrichment is the percentage of genes loaded divide by the percentage of genes with this term in the background. The p-value measured the Fishers exact test. (DOCX) [file pntd.0011516.s003.docx]

**Tables S3:** Significant (p<0.05) gene ontology (GO) enrichment of T.b. rhodesiense biological processes of differentially enriched genes (DEGs) that were upregulated (log2FC > 1) in Rumphi focus and loaded in TritrypDB. The fold enrichment is the percentage of genes loaded divide by the percentage of genes with this term in the background. The p-value measured the Fishers exact test.

| GO ID | Biological Process | Fold enrichment (FE) | P-value of FE |
| --- | --- | --- | --- |
| GO:0075136 | Response to host | 11.47 | 5.53E-05 |
| GO:0052200 | Response to host defenses | 11.47 | 5.53E-05 |
| GO:0052173 | Response to defenses of other organism | 11.47 | 5.53E-05 |
| GO:0051707 | Response to other organism | 11.47 | 5.53E-05 |
| GO:0042783 | Evasion of host immune response | 11.47 | 5.53E-05 |
| GO:0043207 | Response to external biotic stimulus | 11.47 | 5.53E-05 |
| GO:0052572 | Response to host immune response | 11.47 | 5.53E-05 |
| GO:0051701 | Biological process involved in interaction with host | 11.35 | 5.82E-05 |
| GO:0044419 | Biological process involved in interspecies interaction between organisms | 11.24 | 6.11E-05 |
| GO:0044403 | Biological process involved in symbiotic interaction | 11.24 | 6.11E-05 |
| GO:0009605 | Response to external stimulus | 10.18 | 9.77E-05 |
| GO:0009607 | Response to biotic stimulus | 9.86 | 1.14E-04 |
| GO:0019889 | Pteridine metabolic process | 144.58 | 6.90E-03 |
| GO:0050896 | Response to stimulus | 2.92 | 1.27E-02 |
| GO:0015877 | Biopterin transport | 61.96 | 1.60E-02 |
| GO:0042558 | Pteridine-containing compound metabolic process | 48.19 | 2.06E-02 |
| GO:0020033 | Antigenic variation | 8.18 | 2.44E-02 |
