# Supplementary figures and images for: Transcriptome profiles of Trypanosoma brucei rhodesiense in Malawi reveal focus specific gene expression profiles associated with pathology
Source: PLoS Negl Trop Dis. 2024 May 3;18(5):e0011516. doi: 10.1371/journal.pntd.0011516 (PMC11095692; doi:10.1371/journal.pntd.0011516)

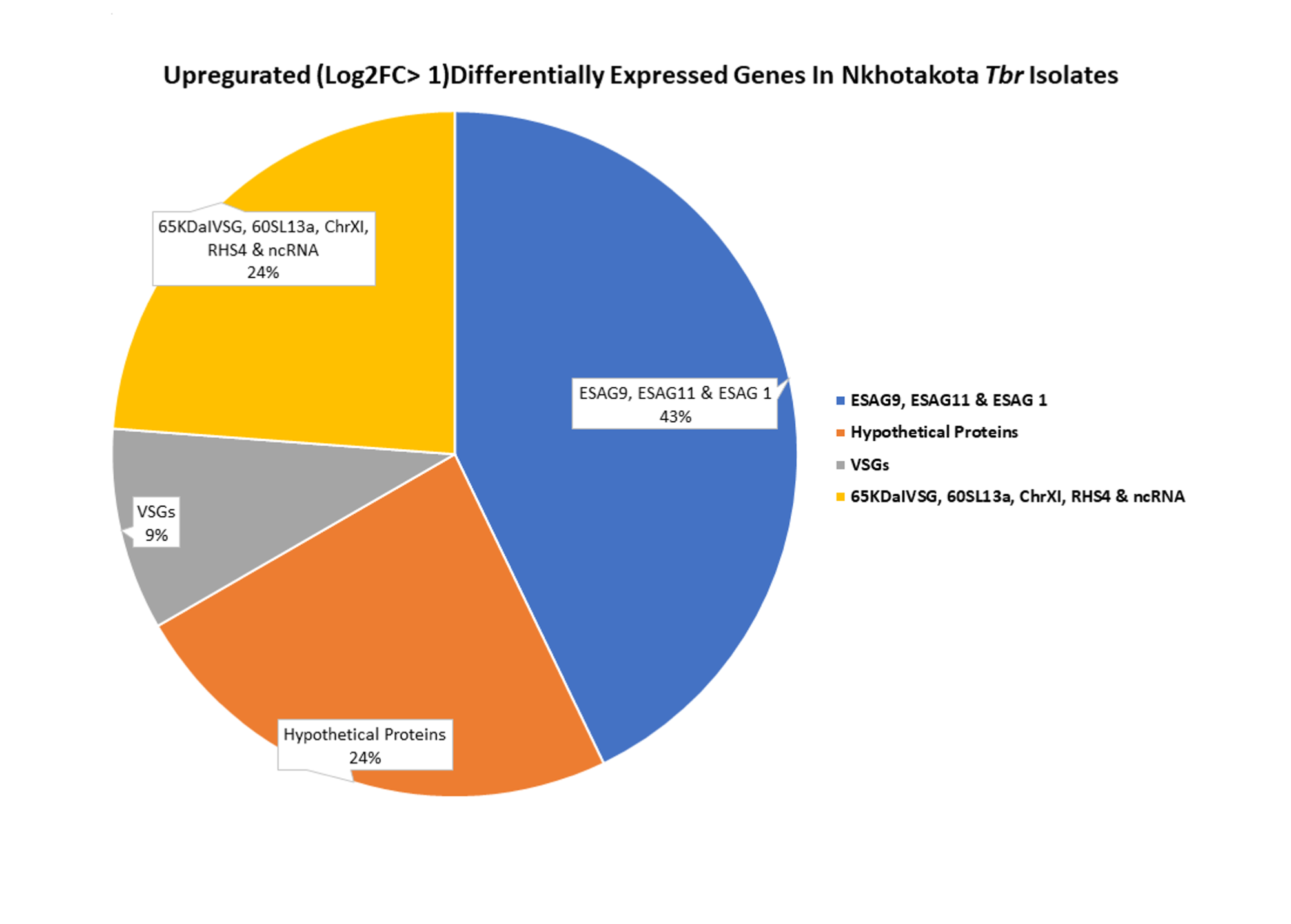

Supplement: S1 Fig — A) PCA analysis showing outlier samples from both Nkhotakota and Rumphi foci that were excluded from further analysis. B) Proportions of upregulated differentially expressed genes in in Nkhotakota Tbr isolates with ESAG transcripts being the most upregulated. (TIFF) [file pntd.0011516.s004.tiff]

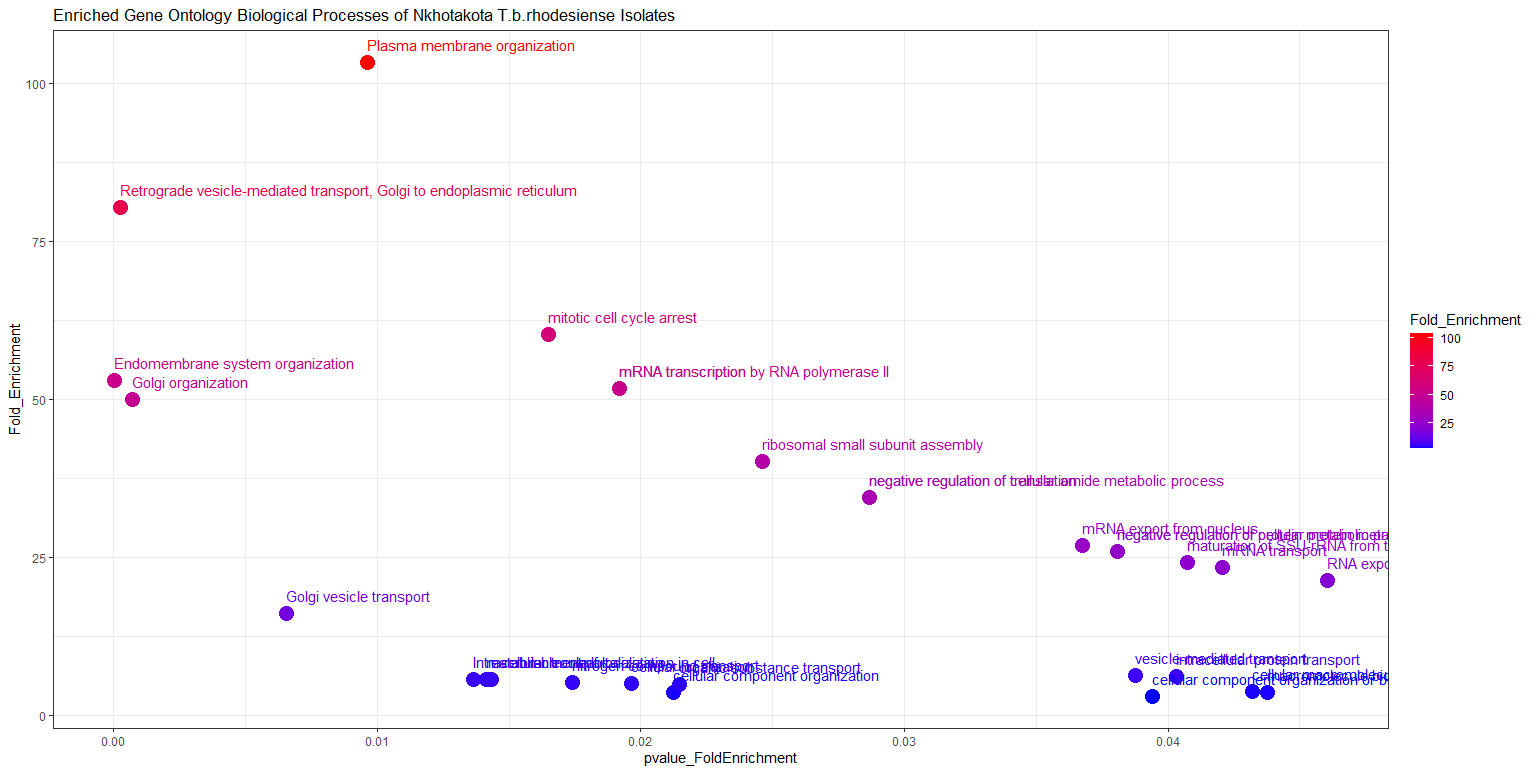

Supplement: S2 Fig — (TIFF) [file pntd.0011516.s005.tiff]

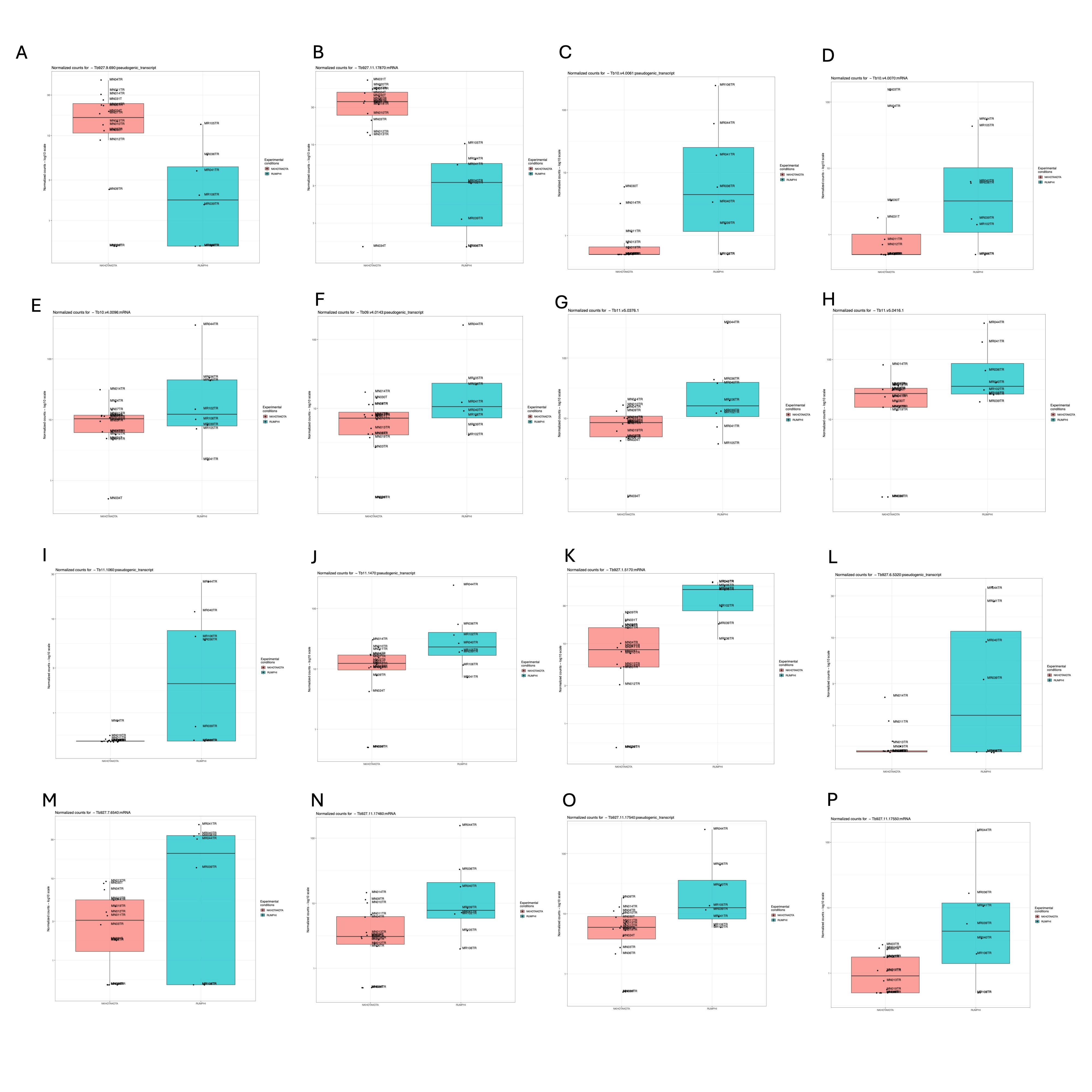

Supplement: S4 Fig — A to B) Normalised counts of VSGs differentially expressed in isolates from Nkhotakota foci. C to P) Normalised counts of VSGs differentially expressed in isolates from Rumphi foci. (JPG) [file pntd.0011516.s007.jpg]
