# Supplementary material for: Transcriptome profiles of Trypanosoma brucei rhodesiense in Malawi reveal focus specific gene expression profiles associated with pathology
Source: PLoS Negl Trop Dis. 2024 May 3;18(5):e0011516. doi: 10.1371/journal.pntd.0011516 (PMC11095692; doi:10.1371/journal.pntd.0011516)
Supplement: Attachment — Submitted filename: Plos NTD review August 2023.docx [file pntd.0011516.s008.docx]

**Review**

**Transcriptome profiles of T.b. rhodesiense in Malawi reveal focus specific gene**

**expression Profiles associated with pathology.**

Major comments

1. For the analysis and especially for the biological pathways, it seems the authors only considered upregulated genes. Why did they ignore the down-regulated genes? Could those also be somehow important in defining the pathology as observed?
2. L309. Authors indicated that all expressed VSGs that were identified by other authors ,Jayaraman et.al., through long read sequencing of T. brucei passages in mice (26), were not differentially expressed in our data and vice versa. This might explain the challenge that has been in identifying expressed antigens that may be used as novel biomarkers for diagnostics and vaccine development in humans. However, its long known that the VSGs are likely not to be a viable option for especially vaccines due to the elaborate antigenic variations that can occur in a natural infection. Thus the difference maybe also as a result of variations in the repertoire to VSGs that is expressed at that point in time.
3. L322 -324: The authors indicate here that “In animal trypanosomes, a unique VSG expressed throughout infection has been used to develop a vaccine candidate that offered protection against T. vivax infection in mice models which has a potential of a clinical trial (27).” The paper that has been referenced showed data for an invariant flagellum antigen and not a VSG as indicated by the authors. Also, the data in that paper showed that there seems to be no immune response targeted against the antigen in a natural T.vivax infection. So I don’t think the antigen in its current form has a potential of a clinical trial.

Minor comments and suggestions

1. L164-166: Authors indicated that 43 % (9/21) of the genes encode for ESAG1, ESAG9 and ESAG11. But It’s not clear whether more than on gene encodes for the 3 ESAGS mentioned here.
2. L250 – The sentence as it is written do not communicate the data to the reader.
3. L255-257 – The authors indicate here that There was a mean Fst of 0.31 between the two populations and 352 SNPs had an Fst of 1.0 of which most were on chromosome 1 (Fig 4C). BUT fig 4 does not have 4c. Can the authors clarify this and make the necessary corrections.
4. L271 – 273 - Identification of kinesin K39 in T. b. rhodesiense isolates highlights this as a potential biomarker for a much need serodiagnosis of T. b. rhodesiense infections and should be validated in future studies. This I believe should be Identification of kinesin K39 in T. b. rhodesiense isolates highlights this as a potential biomarker for a **much needed** serodiagnosis of T. b. rhodesiense infections and should be validated in future studies.
5. L309: The sentence is “For example, all expressed VSG that were identified by Jayaraman et.al.,…” but should be “For example, all expressed **VSGs** that were identified by Jayaraman et.al.,…”
6. L334: change “form each focus” to “from each focus”
