# Supplementary material for: Transcriptome profiles of Trypanosoma brucei rhodesiense in Malawi reveal focus specific gene expression profiles associated with pathology
Source: PLoS Negl Trop Dis. 2024 May 3;18(5):e0011516. doi: 10.1371/journal.pntd.0011516 (PMC11095692; doi:10.1371/journal.pntd.0011516)
Supplement: Attachment — Submitted filename: Response_to_Reviewers.docx [file pntd.0011516.s009.docx]

**RESPONSE TO REVIEWERS COMMENTS**

**Transcriptome profiles of T.b. rhodesiense in Malawi reveal focus specific gene**

**expression Profiles associated with pathology.**

Major comments

1. For the analysis and especially for the biological pathways, it seems the authors only considered upregulated genes. Why did they ignore the down-regulated genes? Could those also be somehow important in defining the pathology as observed?
   - **We apologies for the confusion. Since we compared two Nkhotakota and Rumphi foci and vise versa, gene that were downregulated in Nkhotakota isolates were upregulated in Rumphi isolates. So we have analyzed both upregulated and down regulated genes.**

**The analysis emphasized gene expression differences in isolates that were focus specific, that is Nkhotakota and Rumphi. As shown in Table S1, significant genes which were upregulated isolates from Nkhotakota were interestingly downregulated in the Rumphi isolates. Therefore we did not ignore but rather highlighted the upregulated gene pathways in isolates from one foci which downregulated in the other.**

2. L309. Authors indicated that all expressed VSGs that were identified by other authors ,Jayaraman et.al., through long read sequencing of T. brucei passages in mice (26), were not differentially expressed in our data and vice versa. This might explain the challenge that has been in identifying expressed antigens that may be used as novel biomarkers for diagnostics and vaccine development in humans. However, its long known that the VSGs are likely not to be a viable option for especially vaccines due to the elaborate antigenic variations that can occur in a natural infection. Thus the difference maybe also as a result of variations in the repertoire to VSGs that is expressed at that point in time.
   - **Thank you for this insight. We have removed the sentence “This might explain the challenge that has been in identifying expressed antigens that may be used as novel biomarkers for diagnostics and vaccine development in humans”.**
3. L320 -322: The authors indicate here that “In animal trypanosomes, a unique VSG expressed throughout infection has been used to develop a vaccine candidate that offered protection against T. vivax infection in mice models which has a potential of a clinical trial (27).” The paper that has been referenced showed data for an invariant flagellum antigen and not a VSG as indicated by the authors. Also, the data in that paper showed that there seems to be no immune response targeted against the antigen in a natural T.vivax infection. So, I don’t think the antigen in its current form has a potential of a clinical trial.
   - **We thank you for highlighting this, we have revised the sentence: 1) We have removed “which has a potential of a clinical trial” (Line 323-324); 2) We have replaced “a unique VSG expressed throughout infection” with “a cell surface protein localized to the flagella” (Line 324).**

**We have rephrased the sentence to . “In animal trypanosomes, a cell surface protein localized to the flagella has been used to develop a vaccine candidate that offered protection against *T. vivax* infection in mice models”**

- **We agree with the reviewer that the data we referenced (33) was in rodent experiments not natural infection. However, the use of a cell surface glycoprotein (which was a fragment of *T. vivax* VSG) localized on the flagella highlight it as a potential vaccine candidate against natural T. vivax infections. On the other hand, this study by the authors, Jayaraman et.al., was based on the hypothesis that “the VSGs may also subvert natural immunity by preventing the acquisition of high-titre antibody responses to protective antigens”.**

Minor comments and suggestions

1. L164-166: Authors indicated that 43 % (9/21) of the genes encode for ESAG1, ESAG9 and ESAG11. But It’s not clear whether more than on gene encodes for the 3 ESAGS mentioned here.
   - **We have revised and added the gene IDs for the ESAGs** **(Line 167 to 170).**
2. L250 – The sentence as it is written do not communicate the data to the reader.

- **We have revised the sentence to “To validate the genetic distance in the phylogenetic tree, we used fixation index (Fst)…..” (Line 252-253)**

3. L255-257 – The authors indicate here that There was a mean Fst of 0.31 between the two populations and 352 SNPs had an Fst of 1.0 of which most were on chromosome 1 (Fig 4C). BUT fig 4 does not have 4c. Can the authors clarify this and make the necessary corrections.
   - **Apologies for this error as there is no Fig 4C. We have revised the sentence to “There was a mean Fst of 0.31 between the two populations and 352 SNPs had an Fst of 1.0” (260).**
4. L271 – 273 - Identification of kinesin K39 in T. b. rhodesiense isolates highlights this as a potential biomarker for a much need serodiagnosis of T. b. rhodesiense infections and should be validated in future studies. This I believe should be Identification of kinesin K39 in T. b. rhodesiense isolates highlights this as a potential biomarker for a **much needed** serodiagnosis of T. b. rhodesiense infections and should be validated in future studies.
   - **The sentence has been revised (Line 275).**
5. L309: The sentence is “For example, all expressed VSG that were identified by Jayaraman et.al.,…” but should be “For example, all expressed **VSGs** that were identified by Jayaraman et.al.,…”
   - **This sentence has been removed as suggested by reviewer 3.**
6. L334: change “form each focus” to “from each focus”
   - **This has been revised (Line 335).**

Reviewer #1:

7. The objectives are clear and the hypothesis testable. Since the study essentially examines two spatially seprated populations (Nkhotakota and Rumphi foci), more details on descriptions of these populations and a spartial map where the samples were collected and probably barriers between these 'populations' would help. Secondly, it not clear from the writing on how many replicates were used in RNA Seq... where 16 and 8 cases were collected from Nkhotakota and Rumphi respectively considered as independent replicates? This needs to be clarified. For the population genetics component, the numbers in each population is not clear. Typically a sample size of 30 and above is considered sufficient. More details is required here.
   - **To avoid repetition of already published data within this manuscript, we referred in Line 105 to 106 to our r-HAT surveillance paper by Nambala et.al., 2021 (reference number 2). In this paper we show the spartial map highlighting Nkhotakota and Rumphi foci where the samples were collected and as well describe the populations of these 2 foci. We have added a sentence describing the distance, “The samples were collected from Nkhotakota and Rumphi r-HAT foci which are approximately 400 Km apart” (Line106-107).**
   - **Due to cost implications that were beyond the budget for this study, we could not sequence the samples in replicates. As such we have revised and indicated that each sample was considered as an independent biological replicate for RNA-seq (Line132 to 133).**
   - **We have revised and added the number of samples used for population genetics in the methodology section, “The GATK workflow was used for SNP calling from reads that were mapped to the *T. brucei* transcriptome for 16 samples from Nkhotakota and 8 samples from Rumphi foci” (Line 137 to 138). We have also added a limitation of the sample size we have used for the population genetics (Line 349 to 351).**
8. Appropriate data collection and analyses approaches were employed and ethical approval obtained. The quality of the figures need improvement. Line 186 -"..genes were upregulated (padj> 1.0)...." padj should be <0.05 for significance. The data has not also been deposited in EGA or any other public repository yet, which in turn limits efforts of validating the findings.
   - **We have revised and improved the quality of the figures**
   - **The phrase "..genes were upregulated (padj> 1.0)” has been revised to "..genes were upregulated (log2FC> 1.0)”.**
   - **The phenotype and RNA sequence data was submitted to EGA and so we have included a section on Data availability (line 360).**

Reviewer #3:

9. Line 159-160: “Four samples were identified as outliers in the PCA and were excluded from the analysis (Fig S1A).” Can the authors clearly mark the outliers in Fig S1A and justify why they are considered outliers and not the real representation of the population diversity?
   - **We have reanalyzed the data and included all the 24 samples (16 samples from Nkhotakota and 8 samples from Rumphi, Fig 1A). The 4 samples that were excluded were MR044TR, MR039TR, MN06TR and MN07TR are now shown in Fig 1A. Inclusion of the four samples didn’t affect the number of DEGs nor their expression levels (Table S1).**
10. Lines 165 – 171: On ESAGs being DE: one must be careful inferring from these results because ESAGs belong to multicopy gene families often characterized by redundant genes. So, whilst the upregulation of particular non-redundant ESAG copies may be of relevance, upregulation of redundant ESAGs may just be the product of chance. My suggestions to address this issue are either to acknowledge this limitation in text, or to perform a more in-depth analysis of the DE ESAGs (by phylogenetics for example) to assess whether they are likely to be functionally redundant or not.
    - **We acknowledge this limitation and we have added a limitation statement (Line 351 to 353).**
11. Line 171 and 187: VSGs being DE may just mean that the two populations were expressing different superabundant VSGs at the time of collection. Were these VSGs equally abundant across all samples of the same group? Or is one sample contributing more than others?
    - **We have added Fig S4 that shows the normalized count of significant differentially expressed VSG in each sample. Figure legend is on Line 610-612.**
12. Fig 1B and Fig 2A are the same, just flipped. I suggest merging Fig 1 and 2 together, and identifying in Fig 1B which side of the graph represents upregulation in Rumphi and which side represents upregulation in Nkhotakota. It would also help to plot Log2 FC against average expression, side by side with Fig. 1B.
    - **We have merged the Fig 1B & 2A (Line 391) into Fig 1B and renamed it “Significally Expressed Genes (padj<0.05) in Human *T. b. rhodesiense* isolates in Malawi”**
    - **We have changed Fig 1C to 2A (Line 391).**
13. Line 219: If rodent samples were not included in the analysis, why add them in the first place? It confuses the reader.
    - **We have rephrased the sentence. Most studies on T. brucei rhodesiense have been using isolates in rodents rather than endemic human samples. We believe this information is important to the readers so that they should be cautious when inferring the biology of T. b. rhodesiense in humans using rodent experiments.**
14. Fig 3B: What are the labels of each sample? I recommend adding information on the origin of each sample as in the PCA plot.
    - **We have revised and added the origin of each sample (Line 473-474).**
15. Lines 221-224: The number of DE genes don’t seem to add up. The authors mention a total of 3132 DE genes, but only 1565 upregulated and 753 downregulated in Malawi, leaving 814 genes missing.
    - **We have revised and indicate that 814 genes were neither upregulated nor downregulated (Line 229-230).**
16. Lines 226-228: are those genes highly upregulated also highy abundant? Small changes in genes expressed at very low levels result in very high fold changes, not necessarily relevant.
    - **We have revised and removed this sentence to avoid confusion to the readers.**
17. Fig 4C is not present in my version of the manuscript and therefore I could not review it (referred in lines 255-257).
    - **We apologise for this confusion. This has been corrected (Line 260) and we removed Fig 4C not to be part of the manuscript as it was a redundant figure.**
18. All figure legends need further detail.
    - **We have revised and added further details to all figure legends (Line 393 to 399, 430-434, 473-474 and 493-496)**
19. Line 264: VSGs were not overexpressed, but rather upregulated. This needs to be confirmed as it would only be relevant if their expression level (ultimately represented by read counts rather than fold change) is high enough.
    - **We have revise to “Upregulation of VSGs…” (Line 267).**
20. Line 267: stumpy forms do express VSGs. However, due to their cell arrest state, they have overall much lower leves of mRNA.
    - **Thank you for this insight, we have revise the sentence to “……whereas, stumpy trypanosomes have much lower levels of VSG expression” (Line 269-270).**
21. Line 309-311: one would not expect to find the same VSGs expressed in different samples, especially not in samples from distinct strains, hence the beauty and complexity of antigenic variation. A good example for the situation described in this paragraph would have to be based on an invariant gene and not on a variant antigen.
    - **We have revised and remove the paragraph**
22. Lines 314-317: This is a very interesting finding in the paper. Please elaborate on the expression level of those VSGs and their nature. The authors state they’re unique: does this mean they were never seen in published genomes? What is their degree of nucleotide identity with closest relatives in Tb927? Were they present in the Ugandan samples? Can the authors present the sequences as a supplementary file?
    - **We have removed the word “unique” and revised the sentence to “We have also identified 2 and 14 VSGs in Nkhotakota and Rumphi isolates respectively, that were significant differentialy expressed and upregulated in T. b. rhodesiense in all blood samples analysed” (Line 313-314)**
    - **We have added Fig S4 that shows the normalized counts of each VSG in all samples.**
    - **Thank you for the questions regarding the variantio in these VSGS. However are analyzing these VSGs for another manuscript in writing.**
23. Out of curiosity, how far is Rumphi from Nkhotakota? I’d add this information to the manuscript. It helps to have an idea if a lot of crossover is expected or not.
    - **We have revised and added a sentence that describes the location of Rumphi and Nkhotakota foci. See point number 7 above.**
