# Supplementary material for: Transcriptome profiles of Trypanosoma brucei rhodesiense in Malawi reveal focus specific gene expression profiles associated with pathology
Source: PLoS Negl Trop Dis. 2024 May 3;18(5):e0011516. doi: 10.1371/journal.pntd.0011516 (PMC11095692; doi:10.1371/journal.pntd.0011516)
Supplement: Attachment — Submitted filename: Response_to_Reviewers.docx [file pntd.0011516.s010.docx]

**Response to Reviewers Comments**

A. **Are the figures (Tables, Images) of sufficient quality for clarity?**
1) **Reviewer #2: Authors have sufficiently addressed the concerns in the revised manuscript. However the quality of the figures should be improved as it's difficult to read most of the legends.**

- We appreciate the reviewers concern, we have improved the aspect ratio and legends of the figures to increase clarity in Figure 1A, Fig 2, 3B, 3C and 4A **(Line 393, 425, 458 and 524)**.

B. **Summary and General Comments**
1. **Reviewer #1: while my concerns have been addressed. I am still concerned about the use of single replicate in the analysis.**

We have added a limitation sentence of our analysis “Due to resource limitations, the study used single replicates for RNA sequencing instead of triplicates for control of possible technical variations. Furthermore, it would not be ethical to collect samples at multiple time points prior to treatment, so we will remain dependent on animal studies for understanding the transcriptome over time.” **(Line 350 to 354)**

2. **Reviewer #3: Line 239-244: “A comparison of human Tbr isolates from Malawi and Uganda showed that 3132/7003 (44.72%) gene were significantly (padj< 0.05) differentially expressed of which 1565/3132 (49.97%) gene were upregulated in Malawi (log2FC >1), 753/3132 (24.04%) genes were downregulated, and 814 genes were neither upregulated nor downregulated (Fig 3A and 3B).”

   I appreciate the effort made by the authors, but this is still not correct. 
   If there are 3132 differentially-expressed genes, they must all be either up- or down-regulated. I think the authors are assuming differential expression based on just the adjusted p-value. This is not standard procedure, we should use both LFC and adj p-value as thresholds to consider a gene differentially expressed. Based on the values presented, the total number of DE genes should be 1565+753=2318. The authors should also clearly state the number of transcripts detected in total, which I suppose were 7003, and update the percentages since the total should no longer be 3132.** 

   Thank you for highlighting this. We have revised the percentage for DE genes as recommended to “A comparison of human Tbr isolates from Malawi and Uganda showed that of the 7003 transcripts detected, 3132/7003 genes were significantly expressed (padj< 0.05) in Malawi isolates, of which 2318/3132 (74.01%) were differentially expressed and 814/3132 (25.99%) genes were neither upregulated nor downregulated. Of the differentially expressed genes, 1565/2318 (67.52%) gene were upregulated (log2FC >1) and 753/2318 (32.48%) genes were downregulated (Fig 3A and 3B). **(Line 228 to 233)**

**Line 289: “Slender Trypanosomes use a repertoire of VSGs to evade host adaptive immune system whereas, stumpy trypanosomes have much lower levels of VSG expression” A reference should be added here.**

We have added a reference. **(Line 272)**

**Lines 322-326: “We further propose that T. b. rhodesiense isolates in Nkhotakota focus might be highly transmissible as they overexpressed stumpy markers whereas isolates in Rumphi focus maybe less transmissible due to high maintenance of a slender trypanosome population during human infections.”
As addressed in a previous comment, overexpressed is not the correct term here. Overexpression relates to forced expression in a mutant line. The correct term is upregulation or simply “they increase expression of”.**

- This has been revised to “… they increase expression of…” **(Line 299)**.

**Fig 1B 
-       title: T. b. rhodesiense requires spaces and to be italicized.
-       It’s not clear the directionality of the DE analysis. In other words, which side represents upregulation in Rumphi and which side is in Nkhotakota?
-       Typo in legend: vice-versa**

- Fig 1B legend has been revised accordingly. **(Line 419 to 424)**
